# The Role of the Dental Surgeon in Controlling the Dissemination of COVID-19: A Literature Review

**DOI:** 10.1155/2020/7945309

**Published:** 2020-10-01

**Authors:** Régida C. S. Batista, Caio V. B. Arruda, Marcely Cassimiro, Luciana Gominho, Antônio Carlos Moura, Diana S. Albuquerque, Kaline Romeiro

**Affiliations:** ^1^Dental School, Centro Universitário Facol (UNIFACOL), 85, 55612-650 Vitória de Santo Antão, PE, Brazil; ^2^Department of Dentistry, Dental College of Pernambuco, Universidade de Pernambuco (UPE), 1650, 54753-901 Tabatinga, PE, Brazil; ^3^Department of Restorative Dentistry, Universidade Federal da Paraíba (UFPB), s/n, Cidade Universitária, 58051-900 João Pessoa, PB, Brazil; ^4^Department of Oral Medicine, Real Hospital Português de Beneficência em Pernambuco, 4760-Paissandu, 52010-075 Recife, PE, Brazil

## Abstract

As early as December 2019 in the province of Hubei, China, contamination of patients with pneumonia of an unknown etiology occurred. These patients presented with symptoms such as coughing, sore throat, malaise, diarrhea, high fever, and dyspnea. This emerging disease was named COVID-19 due to being part of the group of coronaviruses (CoVs) belonging to the subfamily *Orthocoronavirinae*, in the *Coronaviridae* family and in the *Nidovirales* order. COVID-19 is most commonly transmitted through speech, coughing, sneezing, and salivary sputum. Because dental professionals work closely with the oral cavity, it is imperative that infection prevention controls are strictly adhered to. It is important that the dental profession treats patients while also limiting the possible contamination through the production of aerosol in the dental environment. Furthermore, the dental professional also has a key role in raising awareness and guidance amongst the population concerning COVID-19 related biosafety measures. This literature review aims to inform dental professionals about the COVID-19 pandemic and to present the implications of the virus to the dentist. Dental professionals are considered to be at high risk for contracting SARS-CoV-2.

## 1. Introduction

The city of Wuhan, in Hubei province, is considered the most populous in China, with a population of over 11 million people. By December 31, 2019, 27 patients with pneumonia of an unknown etiology were identified [1–4]. These patients had clinical symptoms such as coughing, sore throat, malaise, diarrhea, persistent high fever, and dyspnea. Bilateral pulmonary changes were also found in medical imaging exams [5]. Subsequently, the disease was identified as originating from the group of coronaviruses and was given the name COVID-19 by the World Health Organization (WHO) [1, 2]. Initially, several forms of human transmission were speculated to happen, such as oral, fecal, and vertical transmission (from mother to child) [2]. However, its strongest form of transmission is through speech, coughing, sneezing, and salivary sputum [5].

The coronaviruses (COVs) belong to the subfamily *Orthocoronavirinae*, of the family *Coronaviridae* and of the order *Nidovirales* [4–6]. There are currently seven types and COVID-19 is the fourth being a variation of SARS-CoV, and its etiology is associated with bats [7, 8]. The first confirmed cases were associated with the wet live animal markets in the city of Wuhan, where fish and even snakes were being sold [9, 10]. Due to the severity of symptoms presented by those patients, they were hospitalized and underwent imaging and microbiological tests. Subsequently, the Chinese Center for Disease Control and Prevention (on January 10, 2020) publicly shared the gene sequence of the novel coronavirus and completed PCR diagnostic reagent development and testing [3, 10, 11]. To date, COVID-19 has now been detected in 213 countries and territories due to travel and transmission mainly through the expectoration of salivary fluids.

Transmission of COVID-19 via saliva droplets is significant because without precautionary measures, this transmission can occur when there is less than one meter of distance between two people [4]. This directly affects the work of the dental professional as appointments with patients are typically lengthy and the distance between the professional and the patient is minimal [12]. Furthermore, a substantial amount of dental procedures involve the production of aerosols. Therefore, it is believed that the dental professional is one of the most hazardous amongst medical professions when we consider the risk of COVID-19 related contamination [13]. This literature review aims to inform the dental profession about the COVID-19 pandemic and its implications towards the profession of dentistry. In this context, the biosafety rules inherent to the profession are discussed and possible guidelines are presented for a more appropriate dental treatment.

## 2. Materials and Methods

To perform the literature review, the PubMed database was used. Indexed journals that had a correlation with the objective of the study were selected. The following were inclusion criteria for the literature search: COVID-19; SARS-CoV-2; SARS-CoV; Coronavirus; Spittle; Public health; Biosafety; Mouthwash; Infection; Disinfectant in combination with Dentistry. The exclusion criteria for the search included any articles not related to the topic.

## 3. Results

The results obtained after the literature review can be seen in Table 1.

## 4. Discussion

SARS-CoV-2 has a high potential for spreading through saliva, sneezing, coughing, and aerosols expelled through the respiratory tract [2], and it is considered a public health emergency. The knowledge about COVID-19 is limited. However, the disease seems to begin similarly to pneumonia but can then lead the patient to pulmonary fibrosis and even death [4, 25]. The SARS-CoV-2 RNA has a high mutation capacity due to its polymerase proliferative activity. The high mutation rate directly influences the evolutionary potential of the virus. Peng et al. [4] concluded that SARS-CoV-2 has a simple chain genome [26].

### 4.1. SARS-Cov-2 Transmission

Saliva can play a central role in the human-to-human transmission and the development of noninvasive salivary diagnosis can provide a platform for the rapid and early detection of SARS-CoV-2 infection in both a convenient and economical way [13]. Recent reports state that the live virus was present in the saliva of infected individuals [24]. It was also confirmed that the COVID-19 infection happens similarly to that of the SARS coronavirus through the ACE2 cell receptor [27]. SARS-CoV-2 can effectively use the ACE2 as a receptor to invade cells, which can promote human-to-human transmission. ACE2 positive cells have been found abundantly present throughout the respiratory tract and cells morphologically compatible with the salivary gland in the human mouth.

The expression of ACE2 in minor salivary glands was higher than in the lungs which suggest that salivary glands could be potentially targeted by COVID-19 [28]. Saliva samples could be collected from patients who present oropharyngeal secretion as one of the symptoms [29]. Furthermore, it provides the opportunity to determine a diagnosis through a noninvasive saliva sample. This could increase the detection of COVID-19 and reduce the spread of the virus. Initially, the most frequently performed laboratory diagnostic tests are involved with nasopharyngeal, oropharyngeal, and blood samples. Sputum and other samples in severe respiratory illnesses were also considered as samples of the lower respiratory tract [29, 30].

### 4.2. Understanding COVID-19 Spread Cycle in the Dental Clinic

More research is needed regarding the detection of coronavirus in oral fluids and its impact on the transmission of the virus. This will be crucial in improving infection prevention strategies, especially for dentists and other health professionals who perform aerosols-producing procedures. The outbreak of COVID-19 requires the dental professional to take precautions and follow the biosafety measures that prevent the spread of the virus [13].

The transmission of SARS-CoV-2 is the most important concern in dental clinics and hospitals because it is difficult to avoid the generation of large amounts of aerosols, droplets, and even blood mixed with the patient's saliva during clinical practice [15]. Aerosols are solid or liquid particles containing bacteria or viruses, suspended (for at least a few seconds) in a gas [31]. The smaller particles of aerosol (0.5 to 10 *μ*mm in diameter) have the potential to penetrate and lodge in the smaller passages of the lungs and are thought to possess the greatest potential for transmitting SARS-CoV-2 [32]. Therefore, according to Sabino-Silva et al. [13], there is a high risk of transmitting the COVID-19 disease while performing dental procedures because when the operator is working with dental equipment in the oral cavity, such as with high-speed handpieces, there is a significant amount of aerosols generation. Particles, droplets, and aerosols are small enough to remain in the air for a long period before settling on environmental surfaces or entering the respiratory tract.

Potential contamination routes can be bidirectional, meaning the transmission may occur from patient-to-patient, dentist-to-patient, and patient-to-dentist [33] and also between dentists. Thus, the dentist could be an important vector during the pandemic and could promote cross infection. Therefore, the use of personal protective equipment (PPE), such as masks, glasses, visors, chemical and physical protection barriers, constant disinfection of utensils with 70% alcohol, attention to aerosols produced and disseminated by the use of equipment such as high and low speed pens, and ultrasound equipment must be considered. Sattar et al. [27] specified that chemical agents commonly utilized for surface disinfection are tested against SARS-CoV-2 to ensure efficacy.

In this context, health care workers, such as dentists, maybe unaware of how to provide care to patients with asymptomatic infection [29]. Asymptomatic infections seem to be possible [34], and transmission can occur before symptoms of the disease appear. A recent clinical study indicates that 29% of 138 patients hospitalized due to COVID-19 infection in Wuhan (China) were healthcare professionals [35]. As in bronchoscopy, the inhalation of airborne aerosol particles produced during dental procedures in patients with COVID-19 could cause a high risk for COVID-19 exposure to the dental professional. Therefore, it is crucial for dentists to refine preventive strategies to prevent COVID-19 infection, focusing on patient placement, hand hygiene, using PPE, and exercising caution when performing aerosol-generating procedures [36].

### 4.3. Dental Infection Control Practice

According to Qu and Zhou [12], Ji et al. [8], and Guan et al. [11], dental surgeons need to have the knowledge necessary to identify suspected COVID-19 cases. During examination, patients should be asked if they have experienced influenza in the last 14 days, if they have travelled to a highly contaminated area, if they have a fever or were in contact with someone presenting COVID-19 symptoms, in order to interrupt the virus' spread cycle. They should also guide patients and exercise empathy, as the most emotionally fragile patients may develop some oral changes due to the stress caused by the dissemination of false information, which can cause emotional imbalance. These patients may have oral psychosomatic disorders such as dry mouth, burning mouth, atypical toothache, halitosis, bruxism, oral paresthesia, and recurrent aphthous ulcers. These symptoms usually occur due to changes in physical and emotional conditions. In addition, severe SARS-CoV-2 infection along with associated therapy has the potential to contribute to poor oral health such as various opportunistic fungal infections, dry mouth linked to decreased salivary flow, ulcerations, and gingivitis as a result of the compromised immune system and/or susceptible oral environment [19].

According to Sohrabi et al. [5], Peng et al. [4], and Wang et al. [29], dental professionals also have the responsibility of guiding the population on preventive measures to minimize the risks of spreading COVID-19. Simple guidelines should be taught, such as washing hands constantly with soap and water, using 70% alcohol, using alcohol gel, covering your mouth and nose when coughing and sneezing with absorbent paper, being careful in the use and storage of toothbrushes, and maintaining good oral hygiene to control microorganisms.

The pandemic caused by SARS-Cov-2 directly impacts the type of contact between dental professionals and patients [22–24, 26, 32, 33, 37]. This situation has forced dental professionals to postpone nonurgent consultations as a means of safety and perform emergency treatment only. In studies carried out in Germany, contamination of professionals even when the patient had no symptoms was found [3, 4]. Thus, dental care during the pandemic requires even more expressive care with biosafety measures.

At the dental clinic, the whole environment should be considered high risk. Therefore, it is recommended that the dental office provide mask and disinfectant alcohol and that magazines, ornaments, and objects that may spread the virus contagion be removed [17, 38]. Once in the office, the temperature of the patient should be measured with a digital thermometer on the forehead to identify possible fever. If the patient is in an acute febrile state, the appointment should be rescheduled, and the patient should be advised to see a physician [4].

The professional must use PPE appropriately, such as a mask to restrict the spread of fluids, goggles, or face protection and gloves, to avoid cross contamination. Chemical and physical barriers must be properly used, and no appointments should be scheduled for nonurgent procedures, especially in patients with the flu or similar symptoms. Also, biological waste and contaminating materials must be properly disposed of [22, 39].

Before and after the procedures, the professional must be very careful with the risks inherent to cross contamination, since the use of masks does not effectively protect from particles below 05 nm. It is well established that surgical masks do not provide sufficient protection against airborne transmission. The European Standard [EN 149:2001] classifies respirator masks into three different categories: filtering facepiece FFP1, FFP2, and FFP3. The FFP2 is comparable to the US standard N95 [40]. In cases of proven or suspected COVID-19 infection, the safest options are the N95, FFP2 and FFP3 masks because they are more suitable for protection against biological agents. FFP2 and FFP3 have minimum filtration efficiencies of 94% and 99%, respectively [41]. The use of a FFP2/FFP3 (face filtering piece) respirator mask is recommended for all aerosol-generating procedures performed when caring for patients [42].

The use of gowns is also indicated during dental practice. In the USA, ANSI/AAMI PB70 2012 standard classifies surgical and isolation gowns according to their liquid barrier performance with four levels of protection with level 4 offering the most protection against viral and liquid penetration [43]. Level 4 gowns are preserved for high-risk procedures such as surgery or when infectious diseases are suspected according to the American Society for Testing Materials [44]. Thus, for clinical dental care during the COVID-19 pandemic, level 4 gowns are the best choice and should be used. Additionally, nonsterile, disposable patient isolation gowns, which are used for routine patient care in healthcare settings, are also appropriate for use by healthcare personnel when caring for patients with suspected or confirmed COVID-19. In times of gown shortages, surgical gowns should be prioritized for surgical and other sterile procedures [45].

It is noteworthy that the process of removing PPE has the highest risk of contact transfer of viruses from the PPE surface to the skin of healthcare workers [46, 47]. Therefore, it is important to know that the contamination critical zone of a surgical gown comprises the front area of the gown from the chest to the knees and the sleeves from the cuff to above the elbow. The dental professional should be careful not to touch their eyes, nose, and mouth, without proper hand disinfection.

According to Peng et al. [4], the virus is very sensitive to heat and ultraviolet light and can be combated with disinfectant hygiene products, water and soap. According to Kampf [48], Severe Acute Respiratory Syndrome (SARS) coronavirus, Middle Eastern Respiratory Syndrome coronavirus (MERS), or SARS-CoV-2 endemic human coronavirus can persist on inanimate surfaces such as metal, glass, or plastic for up to 9 days but can be effectively inactivated by performing surface disinfection procedures with 62–71% ethanol, 0.5% hydrogen peroxide, or 0.1% sodium hypochlorite for 1 minute [49].

It is also known that the SARS-CoV-2 is not effectively inactivated with chlorhexidine. However, agents such as 1% hydrogen peroxide or 0.2% povidone are recommended to reduce the viral load in the oral environment as they are more effective. Tested povidone-iodine products have strong virucidal activities against SARS-CoV [14], so they have great potential for SARS-CoV-2. However, the combination of chlorhexidine and cetrimide, widely used in human medicine, does not appear to be effective in SARS-COVs, unless ethanol is added. Other mouthwash and/or nasal applications proposed to combat COVID-19 are those that are derived from cyclodextrins combined with Citrox. Together, these compounds could decrease the SARS-CoV-2 viral load and reduce the nasopharyngeal microbiota, which tends to coat the surface of aerosol particles and droplets during coughing or sneezing [18]. This information highlights the need to evaluate the activities of antiseptics and disinfectants against the coronavirus, to ensure its efficiency and develop targeted antisepsis. In addition, the use of intraoral imaging tests should be avoided or performed with great care, as the intraoral radiography device stimulates salivation and pharyngeal reflexes which can induce vomiting. It is indicated to give preference to extraoral radiographic exams or CT scans [3, 29].

According to Meng et al. [2], for a patient who will need urgent treatment, such as a patient with a diagnosis of symptomatic irreversible pulpitis, or painful symptoms due to trauma, the procedure should be performed using absolute isolation and high-volume evacuation. If there is a need for surgery to be performed, preference should be given to absorbable suture threads for soft tissues. If the patient is considered suspected for COVID-19, the dentist should make use of all routine biosafety measures without neglecting any step and make the appointment for this patient either at the end of the work day or provide care in an isolated room to avoid the cross contamination in the office [50].

Overall, any dental procedure that has the potential to generate salivary aerosol could cause airborne contamination of COVID-19. Air purification techniques are needed inside the dental office. As an example, there are HEPA (high-efficiency air) systems, which direct air through a series of prefilters, which help to continuously catch airborne microorganisms and retain particles as small as 0.3 *μ*m to assist in purifying the air in and outside of dental operatories [51] ensuring an environment free of potentially dangerous microorganisms. In the near future, the concept of an ultraclean care room should also be used more in dental offices. In view of the inherent conditions of air contamination by aerosols generated in the dental office, the use of equipment with devices that emit ultraviolet rays, hydrogen peroxide systems, filters, and internal air renewal may soon be a reality in dentistry [52, 53].

## 5. Conclusions

COVID-19 does not have a defined treatment. Dentistry professionals are considered to be at high risk for contracting COVID-19. This is strongly due to COVID-19 transmission through saliva. In light of the high risk of contamination during dental care, only emergency treatment should be performed and PPE such as N95 /FFP2 or FFP3 masks, disposable cloaks, visors, goggles, disposable caps, and gloves should be used. Guidance should be given to patients on maintaining good oral hygiene during the pandemic.

## Figures and Tables

**Table 1:** The potential of SARS-CoV-2 transmission in a dental clinic and measures to prevent and control the infection in dental environments.

Author	Year	Research, results, and conclusions
Kariwa et al. [14]	2006	(i) The use of several PVP-I (Povidone-Iodine) include disinfecting medical instruments and skin as well as hand-washing, gargling, and spraying the throat for coronavirus
		(ii) The results clearly indicate that all the PVP-I products tested have strong virucidal activities against SARS-CoV
		(iii) PVP-I products for gargling and spraying the throat may have a prophylactic effect on SARS during outbreaks

Segal and Wong [15]	2008	(i) Diagnosis through salivary tests is a remarkable process for the discovery of systemic diseases
		(ii) The markers for obtaining diagnoses can be made in the dentist's own office

Cleveland et al. [16]	2016	(i) Measures to prevent and control infections in dental environments must be applied to ensure the safety of patients and professionals, avoiding cross contamination

Ather et al. [17]	2020	(i) It is the duty of the dental surgeon to protect the population and provide quality care even in times of pandemic
		(ii)The dental surgeon must not neglect the biosafety measures in their care. They can assist asymptomatic patients, but even they have a high power of viral transmission
		(iii) Dental professionals are at a high risk of contamination and require more preventive care to avoid the transmission of COVID-19

Carroeul et al. [18]	2020	(i) The use of mouth rinses and/or nasal applications that contain *β*-cyclodextrins, combined with flavonoid agents, such as citrox, could provide valuable adjunctive treatment to reduce the viral load in saliva and nasopharyngeal microbiota, including potential SARS-CoV-2 carriage

Casamassimo et al. [19]	2020	(i) Even in the face of the pandemic, pediatric dentists used ethics to decide whether or not to put themselves at risk to treat the emergency needs of children and special patients
		(ii) Even when performing all biosafety procedures, the risk of contamination is high. This pandemic raises several questions, as dentists are exposed daily to pathogens and infectious agents
		(iii) The pandemic of COVID-19 accelerated the need for the integration of medicine and dentistry. These professionals were recognized for their role in a comprehensive system of care delivery and health behaviours

Coulthard [20]	2020	(i) The pandemic of COVID-19 challenged the entire healthcare system, and health professionals needed to act quickly to find ways to assist those who needed treatment during the pandemic

Chen et al. [1]	2020	(i) SARS-CoV-2 is a newly identified form of coronavirus with significant similarity to SARS-CoV
		(ii) A structural analysis of the receptor binding domain (RBD) of spike glycoprotein responsible for the entry of coronaviruses into host cells through molecular simulation reveals highly similar ternary structures
		(iii) ACE2 is an important receptor for COVID-19
		(iv) Antibodies and small molecular inhibitors that can block the interaction of ACE2 with RBD should be developed to combat the virus

Dziedzic and Wojtyczka [21]	2020	(i) Dental professionals have several challenges in the future after the pandemic of COVID-19, as it is not yet known what oral changes may arise as side effects of the drugs used for treatment. Changes in soft tissues, xerostomia, candidiasis, ulcerations, and gingivitis are some of the possibilities that may occur after the cure of COVID-19

Li and Meng [22]	2020	(i) Dental professionals should be very careful when disposing of personal protection items to avoid cross contamination
		(ii) It is necessary to perform examinations with great care, attempting to avoid or minimize nonurgent care during the pandemic
		(iii) Asymptomatic patients can be sources of transmission of the virus during procedures due to the production of fluids and aerosols

Meng et al. [2]	2020	(i) Everyone should be aware of the risks of infection occurring in dental offices and schools
		(ii) We must maintain the performance of prevention and control measures to avoid postepidemic infections

Peng et al. [4]	2020	(i) Dental professionals are at imminent risk due to the forms of transmission of COVID-19
		(ii) Control and prevention measures against contamination require revising

Prati et al. [23]	2020	(i) Dental procedures have a high potential for contamination for professionals and students in clinical schools
		(ii) Treatments, such as endodontics, which take longer to perform, tend to generate a high quantity of aerosols and oral fluids. Contact between the professional, student, and patient can cause cross contamination

Qu and Zhou [12]	2020	(i) Oral diseases due to psychological stress are indisputable
		(ii) It is the role of the dental surgeon not only to treat the oral health of patients but also to guide and give psychological advice in order to improve the patient's well-being

Sabino-Silva et al. [13]	2020	(i) Prevention measures are necessary for dental professionals due to the mode of viral transmission, occurring by inhalation of aerosols produced by dental procedures

To et al.' [24]	2020	(i) Examination of saliva samples detected coronavirus in 91.7% of patients
		(ii) Saliva examination is promising and noninvasive

Xu et al. [25]	2020	(i) The expression of ACE2 in minor salivary glands was higher than that in the lungs, which suggests salivary glands could be a potential target for COVID-19
		(ii) People with asymptomatic infections could disseminate the virus, as it can concentrate in the salivary glands
		(iii) Saliva is considered a potential focus for the spread of SARS-CoV-2

## Data Availability

No data were used in the study.
